# What’s behind ^68^Ga-PSMA-11 uptake in primary prostate cancer PET? Investigation of histopathological parameters and immunohistochemical PSMA expression patterns

**DOI:** 10.1007/s00259-021-05501-1

**Published:** 2021-08-13

**Authors:** Jan H. Rüschoff, Daniela A. Ferraro, Urs J. Muehlematter, Riccardo Laudicella, Thomas Hermanns, Ann-Katrin Rodewald, Holger Moch, Daniel Eberli, Irene A. Burger, Niels J. Rupp

**Affiliations:** 1 Department of Pathology and Molecular Pathology, University Hospital Zurich, University of Zurich, Zurich, Switzerland; 2 Department of Nuclear Medicine, University Hospital Zurich, University of Zurich, Zurich, Switzerland; 3 Department of Radiology and Oncology, Faculdade de Medicina FMUSP, Universidade de Sao Paulo, Sao Paulo, Brazil; 4 Department of Biomedical and Dental Sciences and Morpho-Functional Imaging, Nuclear Medicine Unit, University of Messina, Messina, Italy; 5 Department of Urology, University Hospital Zurich, University of Zurich, Zurich, Switzerland; 6 Department of Nuclear Medicine, Kantonsspital Baden, Baden, Switzerland

**Keywords:** Prostatic neoplasms, Immunohistochemistry, Glutamate carboxypeptidase II, Positron emission tomography, Neoplasm staging

## Abstract

**Purpose:**

Prostate-specific membrane antigen (PSMA-) PET has become a promising tool in staging and restaging of prostate carcinoma (PCa). However, specific primary tumour features might impact accuracy of PSMA-PET for PCa detection. We investigated histopathological parameters and immunohistochemical PSMA expression patterns on radical prostatectomy (RPE) specimens and correlated them to the corresponding ^68^Ga-PSMA-11-PET examinations.

**Methods:**

RPE specimens of 62 patients with preoperative ^68^Ga-PSMA-11-PET between 2016 and 2018 were analysed. WHO/ISUP grade groups, growth pattern (expansive vs. infiltrative), tumour area and diameter as well as immunohistochemical PSMA heterogeneity, intensity and negative tumour area (PSMA_%neg_) were correlated with spatially corresponding SUV_max_ on ^68^Ga-PSMA-11-PET in a multidisciplinary analysis.

**Results:**

All tumours showed medium to strong membranous (2–3 +) and weak to strong cytoplasmic (1–3 +) PSMA expression. Heterogeneously expressed PSMA was found in 38 cases (61%). Twenty-five cases (40%) showed at least 5% and up to 80% PSMA_%neg_. PSMA_%neg_, infiltrative growth pattern, smaller tumour area and diameter and WHO/ISUP grade group 2 significantly correlated with lower SUV_max_ values. A ROC curve analysis revealed 20% PSMA_%neg_ as an optimal cutoff with the highest sensitivity and specificity (89% and 86%, AUC 0.923) for a negative PSMA-PET scan. A multiple logistic regression model revealed tumoural PSMA_%neg_ (*p* < 0.01, OR = 9.629) and growth pattern (*p* = 0.0497, OR = 306.537) as significant predictors for a negative PSMA-PET scan.

**Conclusions:**

We describe PSMA_%neg_, infiltrative growth pattern, smaller tumour size and WHO/ISUP grade group 2 as parameters associated with a lower ^68^Ga-PSMA-11 uptake in prostate cancer. These findings can serve as fundament for future biopsy-based biomarker development to enable an individualized, tumour-adapted imaging approach.

**Supplementary Information:**

The online version contains supplementary material available at 10.1007/s00259-021-05501-1.

## Background

Prostate-specific membrane antigen (PSMA) is a 100-kDa type II transmembrane protein [1] and is commonly upregulated in prostate carcinoma (PCa) [2]. PSMA expression in PCa correlates with higher tumour grade (Gleason Score) and is an independent predictor for PCa progression [3–5]. Positron emission tomography (PET) targeting PSMA linked to either ^68^Ga or ^18^F has changed imaging approaches for biochemical recurrence and can detect recurrence even on low PSA levels [6] or after focal therapy [7]. Additionally, first prospective studies confirmed an improved PCa staging [8] with focus on detection of nodal or distant disease. Recent data showed improved accuracy for local PCa extension [9, 10], and PCa detection in combination with magnetic resonance imaging (MRI) [11, 12]. It has been suggested that specific prostate cancer tissue features influence PSMA tracer accumulation. About 10% of PCa lack PSMA uptake and cannot be detected by PSMA-PET [13, 14]. In single prostate cancer patients, invisible PCa on multiparametric MRI (mpMRI) but positive ^68^Ga-PSMA-11-PET has been reported [15, 16]. Heterogeneous PSMA expression has been particularly described in metastatic PCa [17], and in PCa with DNA repair defects [18]. It has also been shown that the central zone of the prostate can show false positivity in ^68^Ga-PSMA-11-PET [19]. Furthermore, some reports on PSMA-PET positive lesions that correspond to normal prostatic tissue with increased PSMA expression exist [20]. Only one study correlated immunohistochemical PSMA expression in primary tumours of RPE with corresponding PSMA-PET accumulation, yet [21]. Exact correlation of immunohistochemical PSMA expression patterns in primary tumours of RPE with corresponding PSMA-PET accumulation is mandatory to improve the quality of ^68^Ga-PSMA-11-PET interpretation and to pave the way for optimal molecular imaging in the future.

The aim of this study was to investigate and colocalize immunohistochemical PSMA expression patterns and histopathological features in patients with a pretherapeutic ^68^Ga-PSMA-11-PET followed by radical prostatectomy to identify tumour characteristics that are associated with low SUV_max_ values on PSMA-PET scans.

## Materials and methods

### Study population

This study included consecutive patients who underwent staging with ^68^Ga-PSMA-11-PET for newly diagnosed intermediate or high-risk prostate cancer at the University Hospital Zurich from April 2016 to May 2018. All patients with no radical prostatectomy (RPE) specimen available were excluded. The local ethics committee approved the study protocol (BASEC Nr. 2018–01284) and all patients gave a general written informed consent for use of their data. Relevant clinico-pathological characteristics such as patients’ age at the time of operation, tumour stage, (modified) Gleason Score and WHO/ISUP prognostic grade group were collected.

### Histopathological parameters and Immunohistochemistry

Sixty-two formalin-fixed, paraffin-embedded (FFPE) RPE specimens were evaluated on 2 µm hematoxylin and eosin (H&E)-stained sections. One representative slide from the RPE specimen was chosen for further investigation, harbouring the largest area of tumour and therefore defining the dominant tumour lesion.

Staging and grading were done according to the WHO/ISUP/UICC guidelines [22, 23]. Separate grading of the dominant tumour lesion was done and used for further correlation analysis. The tumour area and maximum diameter on each slide were measured digitally. Very small carcinoma lesions (maximum diameter < 5 mm) were excluded from statistical analysis because of a natural resolution limit of PSMA-PET scans due to partial-volume effects [24].

A newly developed type of growth pattern (infiltrative vs. expansive) of each prostate cancer lesion was determined. We defined infiltrative growth as entrapped benign glands within the carcinoma complexes. An expansive growth pattern showed a tumour infiltration of pure carcinoma glands (without intermingled benign glands) within an area of at least 3 circles of 5 mm^2^ (radius 1.26 mm).

**Fig. 1:**
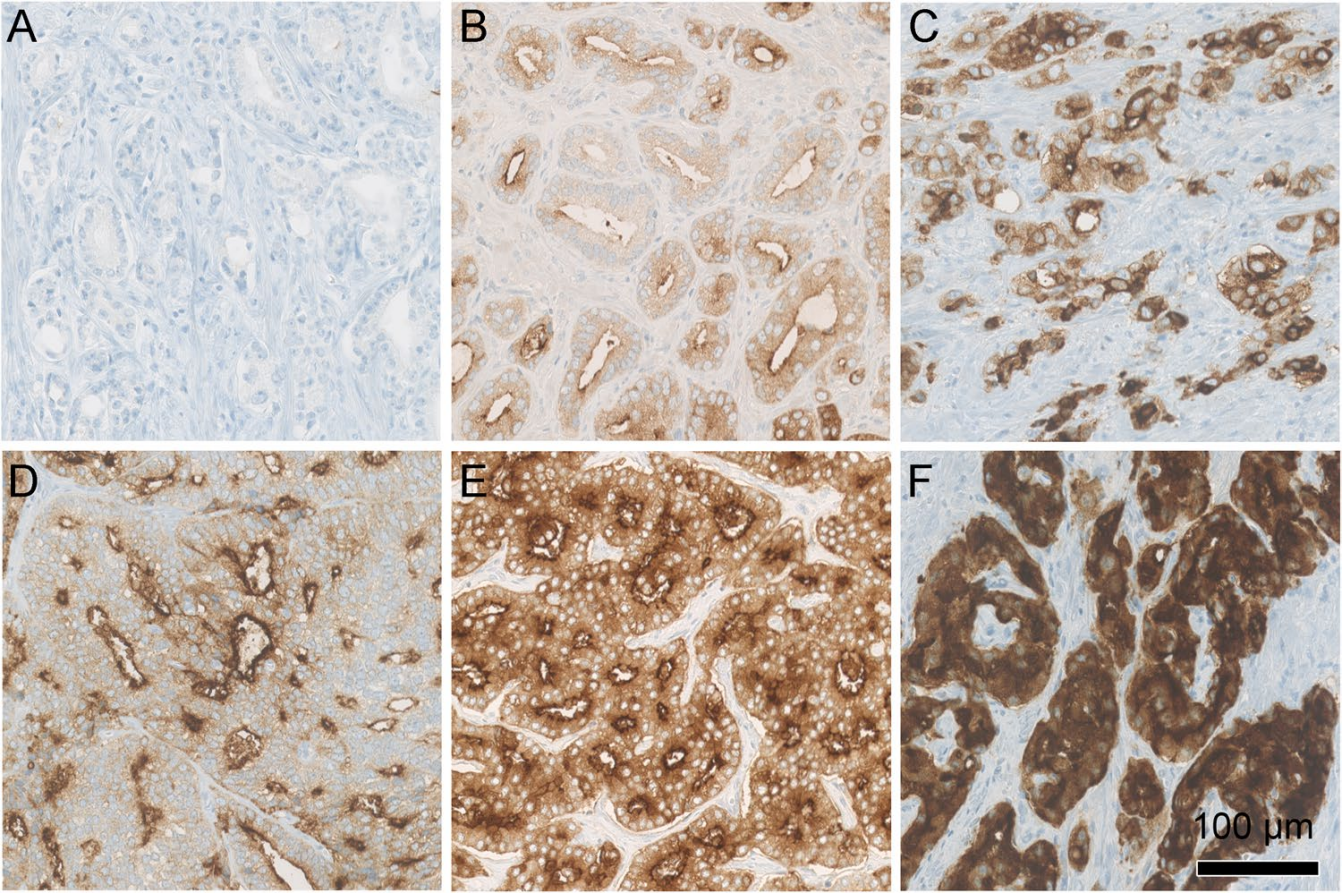
Overview of the different immunohistochemical PSMA staining patterns. (**A**) shows complete negativity, while (**B**) depicts low expression of cytoplasmic (1 +) and moderate membranous (2 +) PSMA staining. In (**C**), a moderate membranous and cytoplasmic (2 +) staining is shown. (**D**) illustrates low cytoplasmic (1 +) and strong membranous (3 +) expression. (**E**) shows moderate (2 +) cytoplasmic and strong membranous (3 +) expression, while (**F**) shows diffuse strong (3 +) cytoplasmic and membranous expression. Scale bar 100 µm

**Fig. 2:**
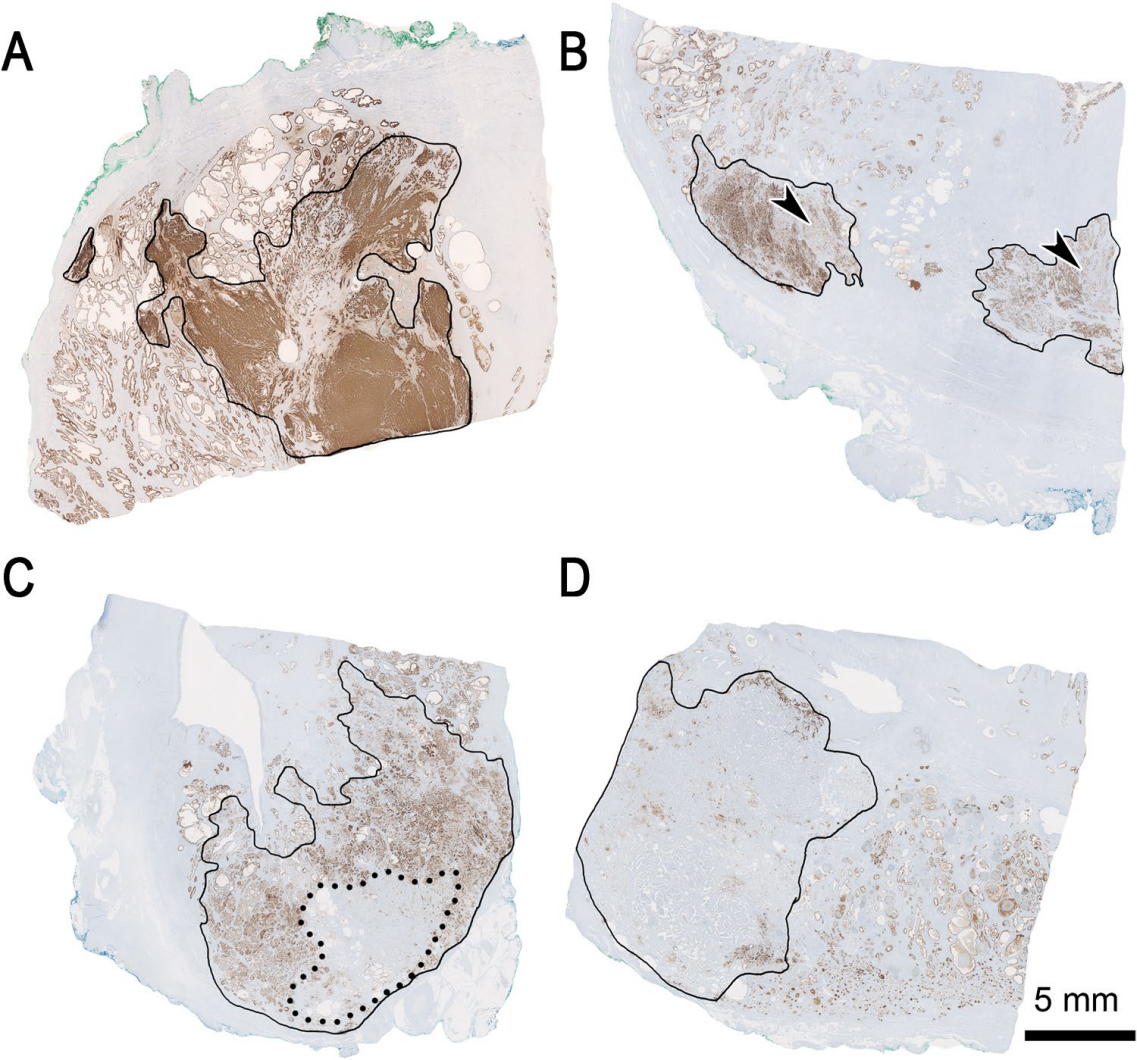
Overview of the different immunohistochemical PSMA heterogeneity patterns. (**A**) shows homogenous strong and diffuse positivity. (**B**) depicts heterogenous PSMA positivity with focal weaker expression (arrowheads) in different components of the carcinoma, without negative areas. In (**C**), the circled carcinoma (continuous line) consists of approximately 30% (dotted line) negative areas, whereas in (**D**), roughly 80% of the marked invasive carcinoma shows negativity. Scale bar 5 mm

Immunohistochemical staining for PSMA (DAKO, M3620, clone 3E6, 1:25) was performed as described previously [25]. The predominant PSMA expression patterns were visually quantified using a four-tiered system (0 = negative, 1 +  = weak, 2 +  = moderate, 3 +  = strong) for both membranous and cytoplasmic PSMA expression by two board-certified, experienced genito-urinary pathologists (J.H.R, N.J.R.). Examples of expression patterns are shown in Fig. 1. Furthermore, tumour areas without PSMA expression were quantified in steps of 5%, 10% and further 10% increments in relation to the total tumour area, as percentage PSMA-negative tumour area (PSMA_%neg_) as a consent of both pathologists. Heterogeneity was defined by differences in the staining pattern of at least 5% of the representative tumour slide (Fig. 2).

Slides were digitalized (Nanozoomer NDP digital slide scanner C9600-12) using the Hamamatsu NDP.view 2.8.24 Software.

### Imaging

Patients underwent clinical routine ^68^Ga-PSMA-11-PET/computed tomography (CT) on a Discovery VCT 690 PET/CT (GE Healthcare, Waukesha, WI, USA) or on a Discovery MI PET/CT (GE Healthcare, Waukesha, WI, USA) or ^68^Ga-PSMA-11-PET/MRI (SIGNA PET/MR, GE Healthcare, Waukesha, WI, USA) after a single injection of ^68^Ga-PSMA-11 (mean dose ± standard deviation (SD) 130 ± 18 MBq, range 81–171 MBq). The institutional protocol is in agreement with the EANM and SNMMI procedure guidelines [26]. Details are given in the supplements.

### Imaging analysis

The acquired PET/CT and PET/MR images were analysed in a dedicated review workstation (Advantage Workstation, Version 4.6 or 4.7, GE Healthcare), which enables the review of the PET and the CT or MR images side by side and in fused mode. Every patient was discussed in a multidisciplinary setup including a pathologist and a nuclear medicine physician and radiologist with the selected pathology slide available alongside with the PET data. The corresponding area on PET images with the dominant tumour lesion was identified and PSMA uptake quantified using the maximum standardized uptake value (SUV_max_). There is a wide range of proposed cutoffs to detect significant prostate cancer from SUV_max_ 3.15 [21] to up to SUV_max_ 9.1 [27]. For visual identification, a clear uptake above background might be more efficient than a absolute cutoff, and given that there were no lesions in the central zone in our cohort, and to select clear positive lesions, we decided to take a PSMA uptake of SUV_max_ ≥ 5 as definition of PSMA-PET positivity [19].

An additional analysis for SUV_max_ ≥ 4 is given in the supplements, to rule out a systematic underestimation (Supplements Fig. S1). Furthermore, PSMA-PET-derived tumour volume was assessed using a fixed threshold at SUV_max_ 4, as well as PSMA-PET tumour to background ratio was calculated and used for correlation with pathology parameters (Supplements Figs. S2-4). For the correlation between immunohistochemical (IHC) parameters and PET quantification, the spatial resolution of the PET scanners was taken into account. Therefore, a tumour diameter of 5 mm or more on histology was considered necessary for accurate quantification of PSMA accumulation limiting the impact of partial-volume effect [24].

### Correlation of histopathological and immunohistochemical parameters with SUV_max_ values

Correlations between histological parameters, immunohistochemical PSMA expression patterns and SUV_max_ values were calculated using the Mann–Whitney *U* test, the Kruskal–Wallis test and Pearson’s correlation. An optimal cutoff for PSMA_%neg_ was determined using receiver operating characteristic (ROC) analysis. We investigated the association between a combination of histological parameters and immunohistochemical PSMA expression patterns with a negative PSMA-PET scan using a multiple logistic regression analysis.

### Statistical analysis

Normal distribution was tested using the Kolmogorov–Smirnov test. Comparisons were calculated with the Mann–Whitney *U* test for binary variables and the Kruskal–Wallis test for multiple variables. Correlations were done using bivariate Pearson’s correlation. Discrimination was evaluated using area under the receiver operating characteristic (ROC) curve (AUC). The variables entered in the multiple logistic regression analysis were selected by univariable logistic regression with a *p*-value cutoff point of 0.05. For the logistic regression analyses, ordinal variables were treated as continuous. Multicollinearity was assessed using variable inflation factors (VIF). To avoid an increase in further parameters, PSMA-PET-derived tumour volume and tumour/background ratios were not included into the multivariant analysis; however, correlation of these parameters with growth patern is given in the supplements. Two-sided *p* values < 0.05 were considered statistically significant. Correlations and ROC curve analysis were performed using SPSS Version 26 (IBM, Armonk, NY, USA). Logistic regression analyses were performed using R (R version 4.0.2; R Foundation for Statistical Computing, Vienna, Austria). Graphs were generated using GraphPad Prism v8.

## Results

### Study population

**Fig. 3:**
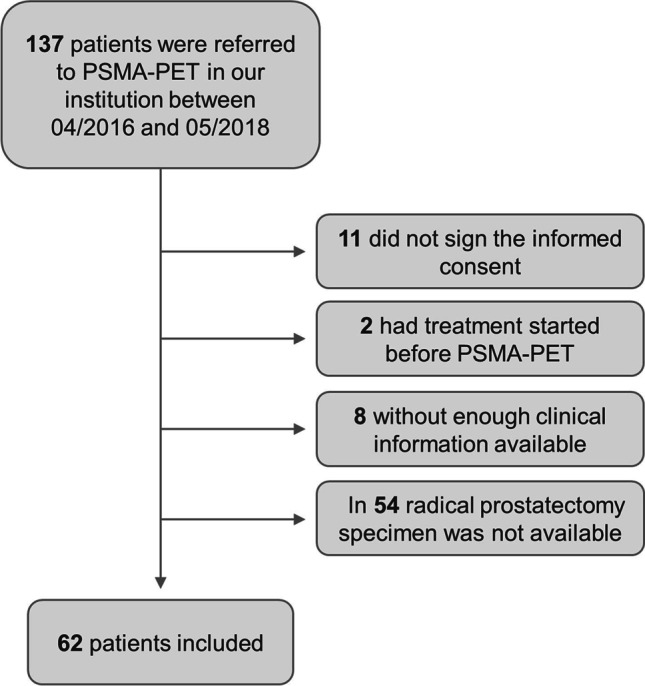
Patient inclusion flowchart

^68^Ga-PSMA-11-PET scans from 137 patients were available. Patients were excluded because of missing informed consent, treatment before PSMA-PET scan, missing clinical information and/or unavailability of a RPE specimen. A total number of 62 patients were included in this study (Fig. 3). Clinico-pathological characteristics are shown in Table 1. Interval between ^68^Ga-PSMA-11-PET and surgery ranged from 1 day to 6 months.

**Table 1:** Clinico-pathological characteristics of the study cohort (*n* = 62)

	*n*/mean	%/SD
Age (years)	63.98	± 6.06
pT stadium	pT2a (*n* = 2)	3.2%
	pT2b (*n* = 2)	3.2%
	pT2c (*n* = 39)	62.9%
	pT3a (*n* = 11)	17.7%
	pT3b (*n* = 8)	12.9%
WHO/ISUP grade groups	Group 2: 3 + 4 (*n* = 5)	8.1%
	Group 3: 4 + 3 (*n* = 23)	37.1%
	Group 4: 4 + 4 (*n* = 21)	33.9%
	Group 5: 4 + 5 (*n* = 13)	21%
Tumour area (mm^2^)	84.3 mm^2^	± 63.5 mm^2^
Tumour diameter (mm)	13.9 mm	± 6.0 mm

### Histopathological parameters

**Fig. 4:**
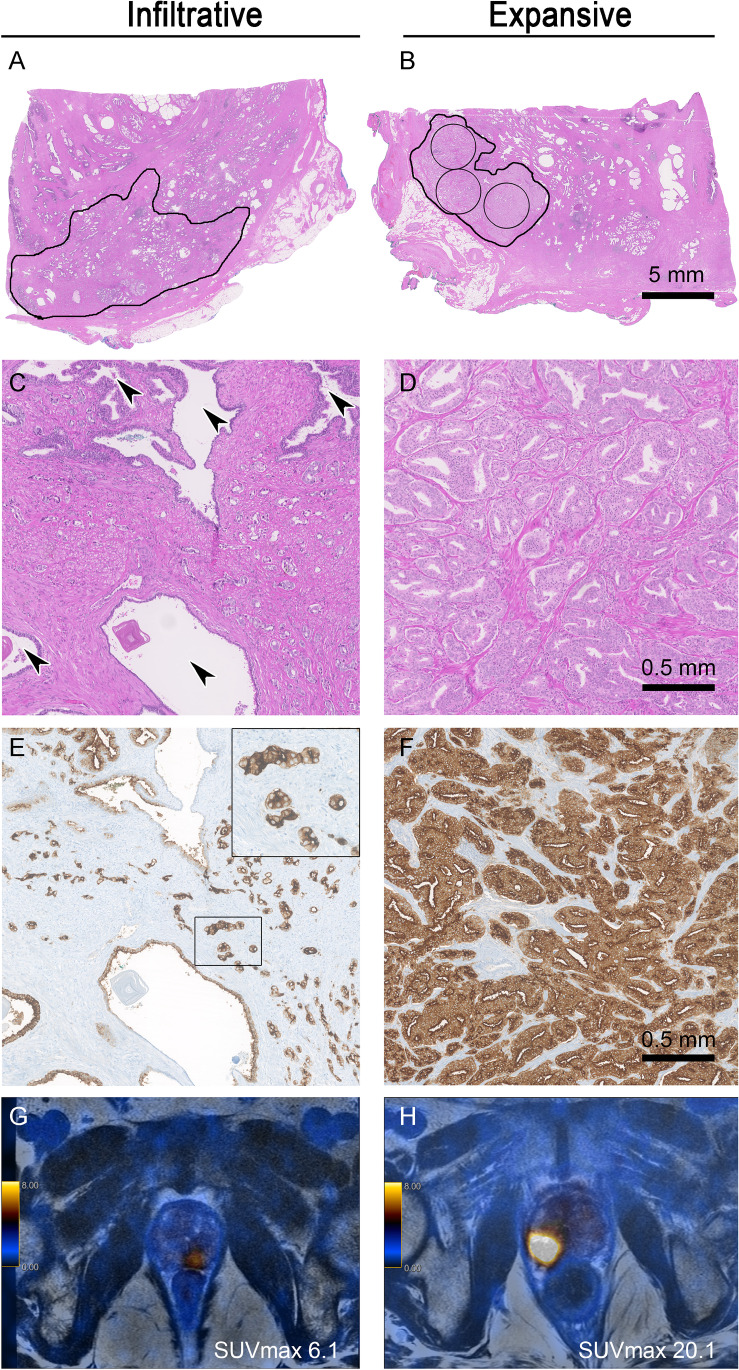
Examples of infiltrative and expansive growth patterns. (**A**, **C**) is an example of a prostate carcinoma growing between normal glands (arrowheads in **C**) refered to as infiltrative growth pattern. (**B**, **D**) depicts a prostate carcinoma which homogenously consists of tumour glands comprising at least 3 circles of 5 mm^2^ each (radius 1.26 mm). This is regarded as expansive growth pattern. While both cases (**A**, **B**) have tumour diameters in a similar range (12 mm and 7 mm, respectively), identical Gleason patterns (both 4 + 4, WHO/ISUP grade group 4) and similar PSMA expression (both cytoplasmic 2 + and membranous 3 +) (**E**, **F**) the SUV_max_ values are clearly different (SUV_max_ 6.1 vs. 20.1) (**G**, **H**). (**A**, **B**) Scale bar 5 mm. (**C**, **D**) Scale bar 0.5 mm

The dominant tumour lesions showed WHO/ISUP grade groups ranging from 2 to 5 (Table 1). The tumour area ranged from 1.4 to 265 mm^2^ (mean 84.3 ± 63.5 mm^2^), and maximum diameter was recorded from 2 to 25.7 mm (mean 13.9 ± 6.0 mm). Four lesions were smaller than 5 mm and were excluded for correlation analysis between histology pattern and PSMA-PET uptake. An infiltrative growth pattern of the dominant tumour was seen in 33 of 62 (53.2%) cases, whereas an expansive pattern occured in 29 of 62 (46.8%) cases (Fig. 4). No significant correlation between growth pattern and WHO/ISUP grade group or pT stage was observed (each *p* > 0.05, Mann–Whitney *U* test). Larger tumour area and higher maximum diameter were significantly correlated with expansive growth pattern (each *p* < 0.05, Mann–Whitney *U* test). Higher WHO/ISUP grade group showed a significant association with higher pT stage (*p* < 0.01, Pearson’s correlation).

### Immunohistochemistry

PSMA expression was noted in all 62 (100%) prostate adenocarcinoma specimen with a range from medium to strong membranous (2 + to 3 +) and weak to strong (1 + to 3 +) cytoplasmic expression (Fig. 1). No case with isolated cytoplasmic without membranous staining was observed. Intratumoural heterogeneity of PSMA expression could be observed in 38 of 62 cases (61%). Twenty-five cases (40%) showed areas completely negative for PSMA comprising 5 to 80% of the tumour area (PSMA_%neg_, Fig. 2).

### Imaging

SUV_max_ values ranged from 3.1 to 48.4 (mean 14.96 ± 10.8). Considering SUV_max_ ≥ 5 as the definition for PET positivity and excluding lesions smaller than 5 mm on histopathology, 49 of 58 scans (84.5%) were positive, and 9 of 58 (15.5%) negative. Of the four lesions smaller than 5 mm, two had a SUV_max_ ≥ 5.

### Correlation of histopathological parameters and immunohistochemistry with SUV_max_ values

The presence of PSMA-negative tumour areas (PSMA_%neg_ between 5 and 80%) was significantly associated with lower SUV_max_ values (mean SUV_max_ 19.24 ± 11.1 vs. 8.89 ± 6.8, *p* < 0.01, Mann–Whitney *U* test).

**Fig. 5:**
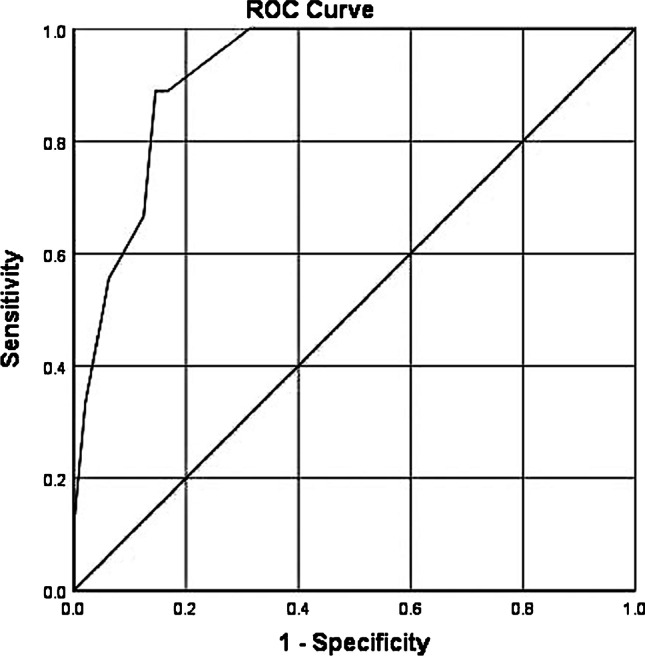
ROC curve analysis for PSMA_%neg_ and negative PET scan. A cutoff value of 20% PSMA_%neg_ yielded sensitivity of 89% and specificity of 86% (area under curve 0.923) for a negative PSMA-PET scan (defined as SUV_max_ < 5)

We performed ROC curve analysis showing the optimal cutoff to be PSMA_%neg_ ≥ 20% resulting in a sensitivity of 89% and specificity of 86% (area under the curve AUC = 0.923) for a negative PSMA-PET scan (Fig. 5).

**Fig. 6:**
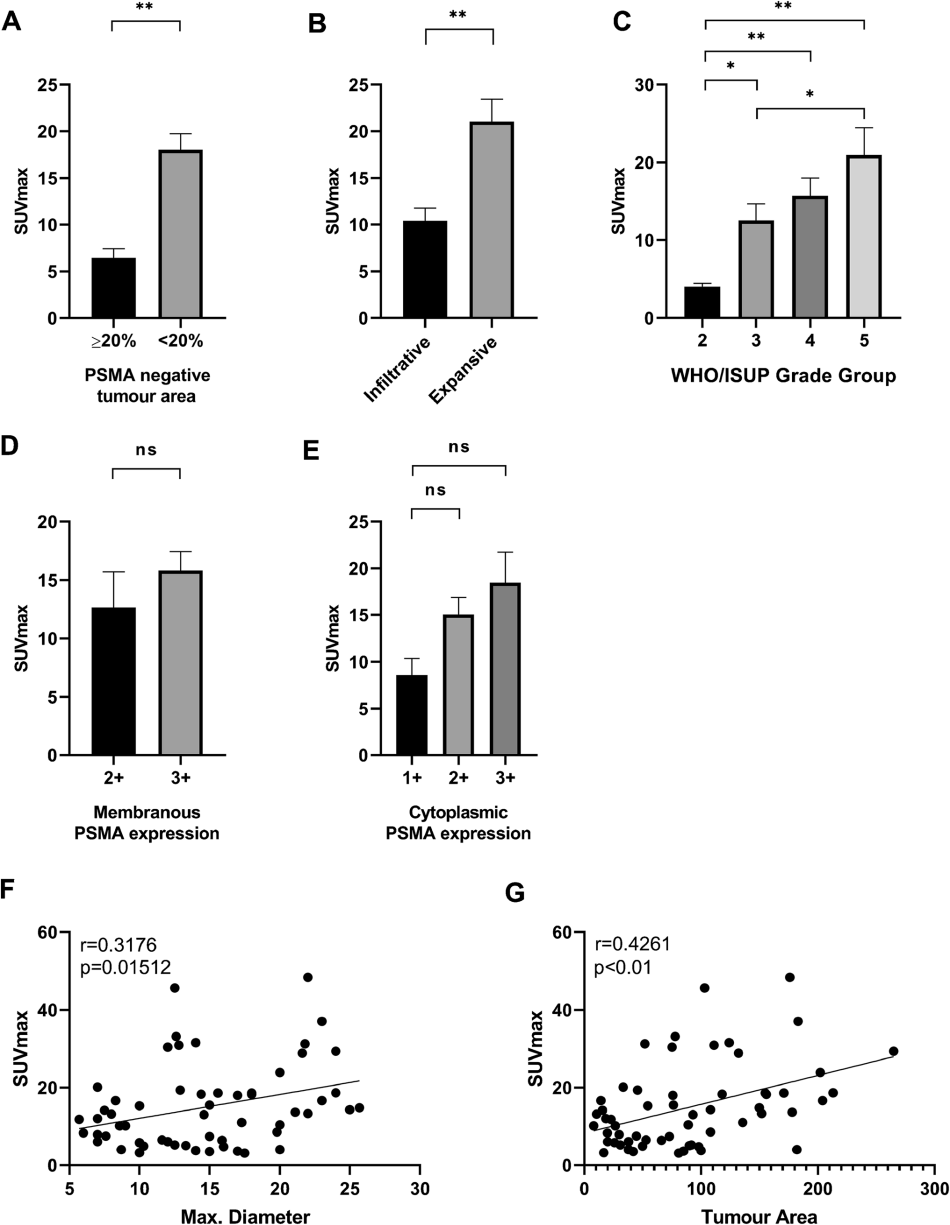
Column plots with standard error of mean (SEM) and scatter plots showing relations between SUV_max_ values and different tissue characteristics. Significant lower SUV_max_ values could be found in prostate carcinomas with PSMA IHC negative area (PSMA_%neg_) ≥ 20% (**A**), an infiltrative growth pattern (**B**), WHO/ISUP grade groups 2 and 3 (**C**) but not in carcinomas with low cytoplasmic or membranous PSMA staining intensities (**D**, **E**). SUV_max_ values significantly correlated with maximum tumour diameter (*r* = 0.318, *p* = 0.015, Pearson’s correlation) as well as tumour area (*r* = 0.426, *p* < 0.01, Pearson’s correlation) (**F**, **G**). *p < 0.05, **p < 0.01

Applying this cutoff (PSMA_%neg_ ≥ 20%) revealed a significant association with lower SUV_max_ values (mean SUV_max_ 17.9 ± 10.9 vs. 6.45 ± 3.7, *p* < 0.01, Mann–Whitney *U* test; Fig. 6A). Eight of nine patients with negative scans had tumours with PSMA_%neg_ ≥ 20% (89% sensitivity). On the other hand, 42 of 49 cases with a PSMA_%neg_ < 20% showed a positive PSMA-PET scan (86% specificity). Infiltrative versus expansive growth patterns showed a significant difference in mean SUV_max_, with expansive tumours having a higher tracer accumulation (mean SUV_max_ 10.0 ± 6.03 vs. 19.9 ± 12.3, *p* < 0.01, Mann–Whitney* U* test) (Fig. 6B). For WHO/ISUP grade groups, a significant difference in SUV_max_ values was observed between group 2 and groups 3 to 5 (*p* = 0.036, *p* = 0.005 and *p* = 0.001, Kruskal–Wallis test) and between groups 3 and 5 (*p* = 0.028, Kruskal–Wallis test) (Fig. 6C). No significant association between cytoplasmic and membranous PSMA IHC expression and SUV_max_ (*p* = 0.11, Kruskal–Wallis test and *p* = 0.13, Mann–Whitney *U* test) was found (Fig. 6D + E). In tumours, which expressed PSMA diffusely (100% positive), homogeneous versus heterogeneous expression was not associated with significantly different SUV_max_ values (*p* = 0.41, Mann–Whitney *U* test). A correlation between WHO/ISUP grade groups and PSMA_%neg_ did not reach significance but showed a trend towards lower grade groups associated with higher percentages of PSMA negative areas (*p* = 0.081, Kruskal–Wallis test). Tumour area and maximum tumour diameter showed a significant positive correlation to higher SUV_max_ values (*r* = 0.426, *p* = 0.001; *r* = 0.318, *p* = 0.015; Pearson’s correlation) (Fig. 6F + G). Additional correlations with tumour volume (Supplements Fig. S2), SUV_max_/SUV_background_ (Supplements Fig. S3) and SUV_max_-SUV_background_ (Supplements Fig.  S4) as well as cribriform growth pattern [28] (Supplements Fig. S5) are reported in the supplements.

PSMA_%neg_, growth pattern, WHO/ISUP grade groups, and cytosolic and membranous PSMA expression were selected as variables for the multiple logistic regression analysis (characteristics of the univariable logistic regression analyses are listed in the supplements). None of the selected variables did show a relevant multicolinearity (VIF < 5). The multiple logistic regression model revealed PSMA_%neg_ (*p* < 0.01, OR = 9.629) and growth pattern (*p* = 0.0497, OR = 306.537) as independent predictors for a negative PSMA-PET scan. Table 2 lists the characteristics of the multiple logistic regression model.

**Table 2:** Results of a multiple logistic regression model to predict negative PSMA-PET scans

Variable	Estimate (log odds)	SE	*p*-value	OR	95% CI OR
Intercept	4.657	4.532	0.304	105.279	0.015–758,328.777
PSMA_%neg_ (per 20% change)	2.265	0.043	0.008	9.629	8.854–10.471
Infiltrative growth pattern	5.725	2.917	0.0497	306.537	1.007–93,274.818
WHO/ISUP grade group	− 2.132	1.205	0.077	0.119	0.011–1.258
PSMA_cytosol_	− 1.345	1.650	0.415	0.261	0.01–6.608
PSMA_membr_	− 1.581	2.225	0.477	0.206	0.003–16.116

*CI*, confidence interval; *OR*, odds ratio; *PSMAcytosol*, PSMA expression in the cytosol; *PSMAmembr*, PSMA expression on the membrane; *PSMA*_*%neg*_, PSMA-negative tumour area; *SE*, standard error

## Discussion

In the present study, we correlated ^68^Ga-PSMA-11-PET results with immunohistochemical PSMA expression patterns as well as histopathological features in prostate carcinomas of 58 RPE specimens using a precise colocalization approach. Significantly lower SUV_max_ values were found in PSMA-PET of prostate carcinoma with PSMA_%neg_, infiltrative growth pattern, smaller tumour size and WHO/ISUP grade group 2. No significant differences in SUV_max_ could be observed regarding cytoplasmic and membranous PSMA IHC expression intensity levels.

**Fig. 7:**
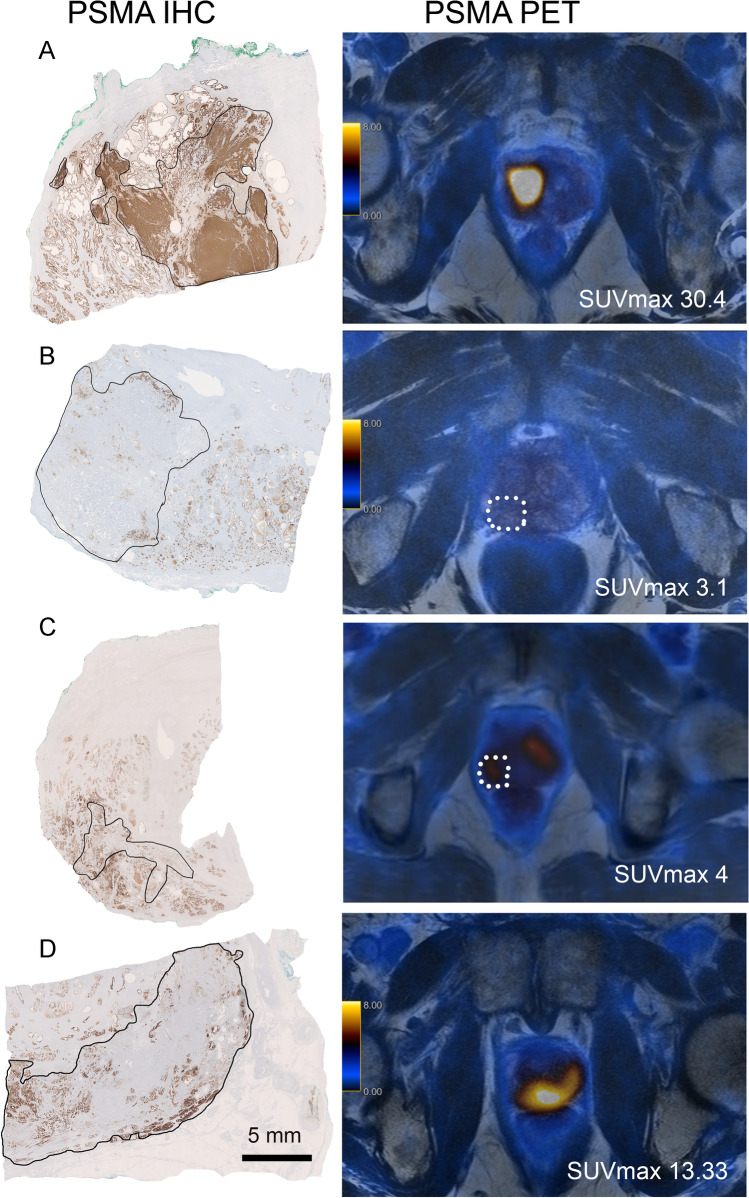
Examples of PSMA expression on immunohistochemistry (IHC) and ^68^Ga-PSMA-PET results. (**A**) Illustration of a prostate carcinoma with a strong homogenous PSMA expression (left) and a high SUV_max_ located in the anterior right part of the gland. (**B**) Example of a prostate carcinoma (circled) showing almost no PSMA IHC staining (80% negative tumour area) and also lacking PSMA-PET positivity in the corresponding area. (**C**) is a case that shows a predominantly PSMA IHC-positive prostate carcinoma (only 5% completely PSMA-negative glands) with a low PSMA-PET positivity (SUV_max_ 4). Note the small diameter of this carcinomatous focus (8.7 mm). (**D**) Conversely, this carcinoma with a diameter of 22 mm shows a high PSMA-PET positivity (SUV_max_ 13.33) despite a heterogenous, largely lacking PSMA IHC expression (almost 70% negative areas). Scale bar 5 mm

Direct correlation of ^68^Ga-PSMA-11-PET uptake with immunohistochemical PSMA expression in RPE specimen has been described only by Woythal et al. They demonstrated a significantly lower SUV_max_ in PCa RPE specimen (*n* = 31) which showed an immunoreactive score (IRS) smaller than 2 or a PSMA staining in less than 50% of the tumour cells [21]. In our analysis, we also confirmed significantly lower SUV_max_ values in PCa showing PSMA negative areas (ranging from 5 to 80%) (Fig. 7A + B). In a ROC curve analysis, we determined an optimal cutoff value of ≥ 20% PSMA_%neg_ yielding the highest sensitivity and specificity for a negative PSMA-PET (defined as SUV_max_ < 5). In terms of PSMA intensity, we scored the cytoplasmic and membranous PSMA expression separately instead of using a score where cytoplasmic and membranous expression is evaluated simutanously (e.g. the IRS). Interestingly, we found only a positive trend of cytoplasmic (*p* = 0.11, Kruskal–Wallis test) and membranous (*p* = 0.13, Mann–Whitney *U* test) PSMA expression levels correlating with PSMA-PET positivity, which did not reach statistical significance.

Although different parameters correlated with PSMA-PET negativity, PSMA_%neg_ and infiltrative growth pattern were found to predict negative PSMA-PET scans in a multiple logistic regression model. This is in concordance with another recently published paper of our group describing PSMA_%neg_ as a parameter able to predict negative PSMA-PET scans in a biochemical recurrence setting [29]. The selection of an absolute cutoff for negative and positive scans is controversial; we therefore selected values with SUV_max_ ≥ 5 for the manuscript, and SUV_max_ ≥ 4 in the supplements, both yielding PSMA_%neg_ ≥ 20% as the optimal cutoff for negative versus positive scans (Supplements).

The investigation of histopathological parameters, such as the growth pattern of the prostate cancer lesion (detached from conventional grading) in exact correspondence to PSMA-PET, has not been published yet. We defined an infiltrative growth as entrapped benign glands within the carcinoma complexes whereas an expansive growth pattern showed pure carcinoma glands within an area of at least 3 circles of 5 mm^2^ each (radius 1.26 mm). In an infiltrative growth pattern, the density of tumour complexes is decreased by intermingled benign glands. As Woythal et al. stated, benign glands not only show lower PSMA expression but also have a significant lower SUV_max_ in the PSMA-PET than prostate carcinoma [21]. Multiple logistic regression also reached statistical significance for infiltrative growth pattern predicting a negative PET scan (*p* = 0.0497).

We decided to exclude all (*n* = 4) very small prostate carcinomas (diameter < 5 mm) because of a natural resolution limit of PSMA-PET scans due to partial-volume effects [24]. Woythal et al. stated no correlation between mean tumour size and SUV_max_. Instead, in this study, a significant correlation between tumour size and SUV_max_ values could be observed. Most likely, this can be explained by different measurement methods. Instead of using the tumour size documented in the pathology report, we measured maximum diameter and area of each dominant tumour lesion on one slide and precisely compared this to PSMA-PET uptake of the corresponding area.

We detected a significant correlation between lower WHO/ISUP grade groups and lower SUV_max_ values. This is well in line with the current literature [3–5]. Additionally, a trend towards higher percentages of PSMA IHC negative tumour areas and lower WHO/ISUP grade groups could be shown.

Looking at deviating cases, the maximum diameter and growth pattern seem to be influential parameters. Only one case with a PSMA negative area < 20% (1 of 43, 2.3%) revealed a SUV_max_ value of 4 (cutoff for a negative PET SUV_max_ < 5). This tumour had a relatively small diameter of 8.7 mm (overall mean 13.9 mm) and showed an infiltrative growth pattern (Fig. 7C). On the other hand, three cases with PSMA negative areas of more than 20% showed high SUV_max_ values of 11, 13.2 and 13.7. All of these cases revealed a relatively large diameter with a mean of 20.1 mm (overall mean 13.9 mm). Moreover, two of them showed an expansive growth pattern (Fig. 7D). The adjacent benign prostate glands in these cases showed an unremarkable, heterogeneous PSMA expression (Supplements Fig. S6).

Our study faces some limitations, including its retrospective and single-centre approach. Furthermore, even in our relatively large cohort consisting of 62 RPEs, the number of cases with a high ratio of negative PSMA areas was limited (15 cases PSMA_%neg_ ≥ 20% and 6 cases with PSMA_%neg_ ≥ 50%) due to the known natural low frequency of PSMA-negative tumours, of around 10% of all PCa. The low number of negative PSMA-PET scans (9 cases) limited the multiple logistic regression analysis. Although evaluations were done by two experienced genito-urinary pathologists, semiquantitative PSMA IHC scoring harbours a certain degree of variability.

This study describes histopathological parameters and immunohistochemical PSMA expression patterns influencing PSMA-PET uptake in RPE specimen. These parameters can be considered as the foundation for potential future biomarkers for PSMA-PET interpretation in prostate cancer. For routine clinical application in a staging setting, these findings need to be transferred to core needle biopsies taken before RPE.

## Conclusion

This study describes immunohistochemical PSMA-negative tumour area, infiltrative growth pattern, smaller tumour size and WHO/ISUP grade group 2 as parameters associated with lower PSMA-PET uptake in RPE specimen of primary prostate cancers. Particularly, 20% or more PSMA_%neg_ showed the strongest association with negative PET scans. Assessment of histopathological parameters and PSMA expression may serve as the basis of future biopsy-based biomarker development for an individualized imaging approach.

## Supplementary Information

Below is the link to the electronic supplementary material.

Supplementary file1 (DOCX 535 KB)

## Data Availability

The datasets used and/or analysed during the current study are available from the corresponding author on reasonable request.
