# Supplementary material for: What’s behind 68Ga-PSMA-11 uptake in primary prostate cancer PET? Investigation of histopathological parameters and immunohistochemical PSMA expression patterns
Source: Eur J Nucl Med Mol Imaging. 2021 Aug 13;48(12):4042–53. doi: 10.1007/s00259-021-05501-1 (PMC8484204; doi:10.1007/s00259-021-05501-1)
Supplement: Supplementary file 1 — Supplementary file1 (DOCX 535 KB) [file 259_2021_5501_MOESM1_ESM.docx]

**Supplementary Material**

**Imaging protocols**

*PET/MR protocol*

A clinical routine whole-body PET/MR was performed 60 min after injection on a hybrid scanner (SIGNA PET/MR, GE Healthcare, Waukesha, WI, USA). PET acquisition for the whole-body protocol was in 3D time of flight (TOF) mode, six bed positions with 2-3 min acquisition time per bed position, an additional pelvic frame over 15 minutes was acquired but not used for quantification, axial FOV of 25 cm and overlap of 24%, matrix 256x256, 2 iterations, 28 subsets, with sharpIR algorithm (GE Healthcare), and 5 mm filter cutoff.

**Table S1: Details of MRI sequences are given in the table below:**

|  | Axial DWI EPI (Focus) (Pelvis) | Axial LAVA-FLEX WB (DIXON) | Axial T1w Whole ARC (Pelvis) | Axial T2w FRFSE-XL  (Pelvis) | Coronal T2w WB FRFSE-XL | Coronal T2w  FRFSE-XL  (Pelvis) | Axial DCE  (Lava Dyn)  (Pelvis) | Ax syn. DWI Focus | Sag T2w  FRFSE |
| --- | --- | --- | --- | --- | --- | --- | --- | --- | --- |
| Repetition time, TR (ms) | 4000 | 5·6 | 550 | 5034 | 5538 | 5034 | 6·361 | 3500 | 4678 |
|  |  |  |  |  |  |  |  |  |  |
| Echo time,  TE (ms) | 67·3 | 1·3-2·7 | 8·26 | 120 | 120 | 120 | 2·376 | Minimum | 120 |
|  |  |  |  |  |  |  |  |  |  |
| Flip angle,  FA (degrees) | 90 | 12 | 111 | 140 | 111 | 140 | 30 | - | 140 |
|  |  |  |  |  |  |  |  |  |  |
| Acquisition matrix | 160 x 80 | 344 x 256 | 384x384 | 300x280 | 288 x 224 | 300x280 | 160 x 80 | 140x70 | 300x272 |
|  |  |  |  |  |  |  |  |  |  |
| Image size (voxels) | 256 x 256 | 512 x 512 | 512 x 512 | 512 x 512 | 512 x 512 | 512 x 512 | 288 x 192 | - |  |
|  |  |  |  |  |  |  |  |  |  |
|  |  |  |  |  |  |  |  |  |  |
| Slice thickness (mm) | 4 | 3 | 5 | 3·5 | 5 | 3·5 | 4 | 4 | 3·5 |
|  |  |  |  |  |  |  |  |  |  |
| Signal averages | 8 | 0·68 | 0·5 | 2 | 0·5 | 4 | 0·35 | - |  |
|  |  |  |  |  |  |  |  |  |  |
| b-values (s/mm2) and signal averages | 0 (6 av.)  400 (8 av.)  700(16 av.) |  |  |  |  |  |  | 0  400  1000  1500  2000 |  |
|  |  |  |  |  |  |  |  |  |  |
| Diffusion direction | ‘All’ |  |  |  |  |  |  | All |  |
|  |  |  |  |  |  |  |  |  |  |
| Bandwidth (Hz/pixel) | 1953 | 166 | 62·5 | 50 | 90·9 | 50 | 62·5 | 250 | 50 |
|  |  |  |  |  |  |  |  |  |  |
| Acquisition time (mm:ss) | 5:41 | 0:18 | 1:44 | 3:27 | 0:50 | 3:27 | 3:27 | 4:05 | 3:12 |

*PET/CT protocol*

Discovery VCT 690 PET/CT (GE Healthcare, Waukesha, WI, USA) and Discovery MI PET/CT (GE Healthcare, Waukesha, WI, USA)

PET was acquired in 3D TOF mode, with six bed positions with 2·5 min acquisition time per bed position, 23% overlap of bed positions, and an axial FOV of 153 mm. The emission data were corrected for attenuation using CT and were iteratively reconstructed (matrix size of 256 x 256 pixels, 3D TOF ordered subset expectation maximization with 3 iterations and 16-18 subsets, with point spread function, 4·7 mm full width at half maximum, 1:4:1 weighted axial filtering). The attenuation CT scan was acquired with whole-body scan parameters as follows: tube voltage 140 kV, tube current with automated dose modulation with a maximum of 80 mA/slice, collimation 512 x 0.976, pitch 0·984:1, rotation time 0.5 s, coverage speed 78 mm/s, field of view (FOV) 50 cm, and images with a transverse pixel size of 0·976 and a slice thickness of 1·25 mm reconstructed in the axial plane.

**Table S2: Variable selection for the multiple logistic regression analysis**

Variable selection for the multiple logistic regression analysis was done by using univariable logistic regression (for ordinal and interval/ratio scaled variables) and Pearson Chi-Square (for nominal scaled variables).

Results of the univariable logistic regression are given in the table below.

| **Variable** | **Significance** |
| --- | --- |
| PSMA_%neg_ | <0.01 |
| Tumour area | 0.498 |
| Tumour maximum diameter | 0.826 |
| WHO/ISUP grade group | 0.035 |
| PSMA cytosolic | 0.023 |

Results of the Pearson Chi-Square given in the table below.

| **Variable** | **Significance** |
| --- | --- |
| Growth pattern | 0.011 |
| PSMA membranous | 0.027 |

**Supplemental figures:**


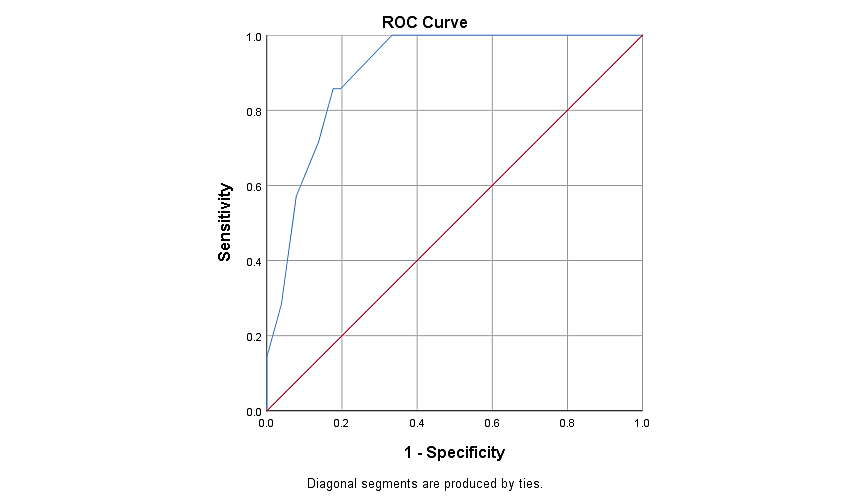


**Figure S1:** **Alternative cut-off for positive PSMA-PET:** To rule out a systematic underestimation due to a cutoff at 5, we also calculated the ROC curve analysis with sensitivity and specify for SUVmax ≥ 4. **ROC Curve analysis** yielding the identical cutoff value of 20% PSMA_%neg_ with a corresponding sensitivity of 86% and specificity of 82% (area under curve 0.905) for a positive PSMA-PET scan **(defined as SUV_max_** ≥ **4)**.

C

B

A


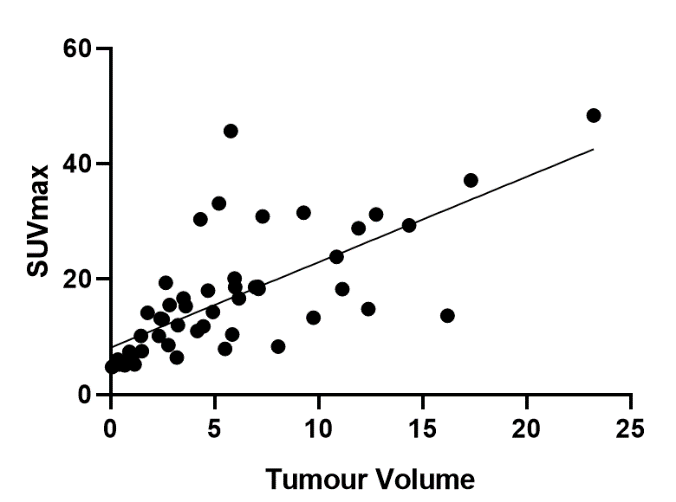

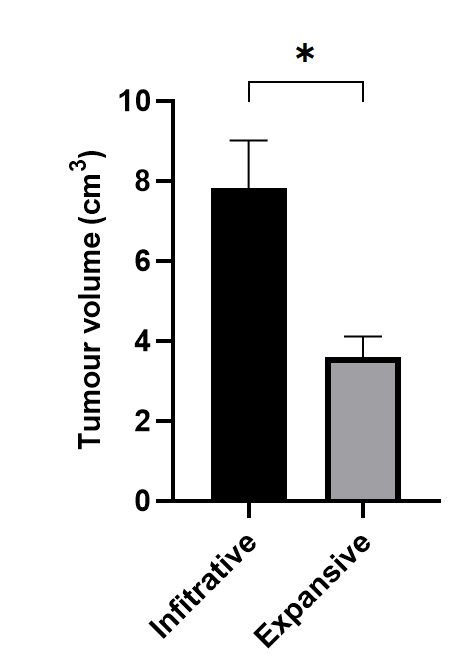

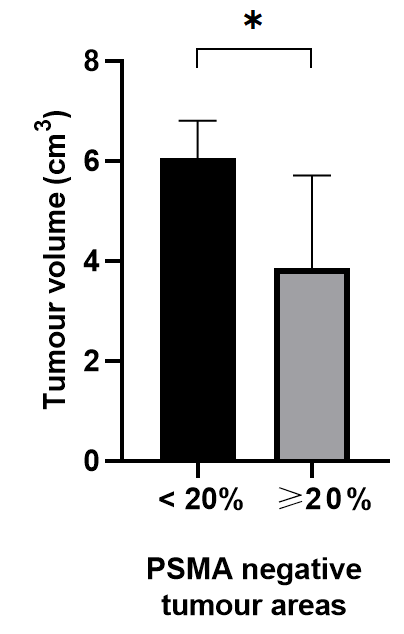


**Figure S2:** **Correlation tumour volume with growth pattern, PSMA negative tumour areas, SUV_max_ values:** Tumour volumes were measured on PSMA-PET scans with an absolute threshold at SUV_max_ 4.

**A)** Significant higher tumour volumes could be found in prostate cancers with an infiltrative growth pattern (p=0.039, Mann-Whitney U test). **B)** Significant higher tumour volumes could be found in prostate cancers with PSMA IHC negative area (PSMA_%neg_) < 20% (p=0.038, Mann-Whitney U test). **C)** Significant correlation between tumour volume and SUVmax values could be seen (r=0.701, p<0.01, Pearson’s correlation).

B

A


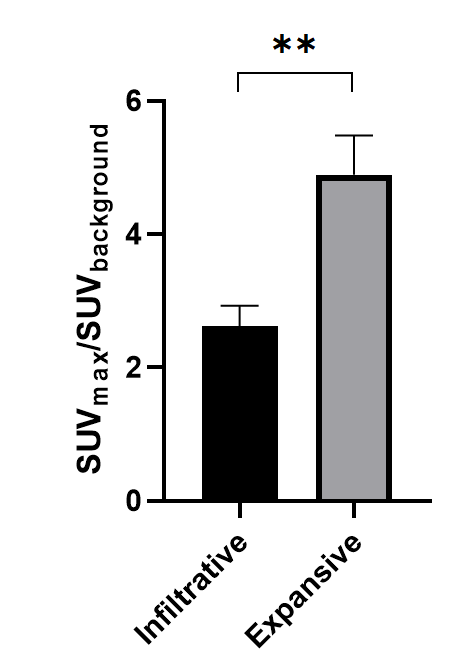

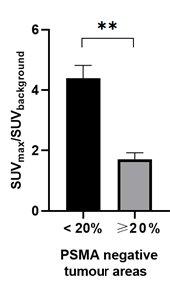


**Figure S3:** **Correlation of SUV_max_/SUV_background_ with growth pattern and PSMA negative tumour areas.**

Significant higher SUV_max_/SUV_background_ ratio were seen in prostate cancers with an expansive growth pattern (p=0.002, Mann-Whitney U test) **(A)** and PSMA IHC negative area (PSMA_%neg_) < 20% (p<0.01, Mann-Whitney U test) **(B)**.

B

A


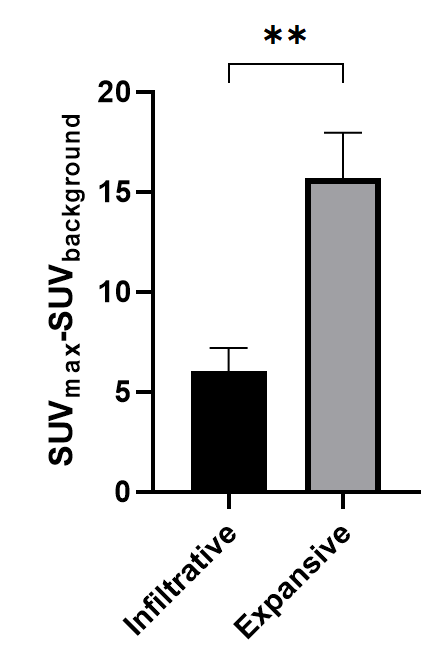

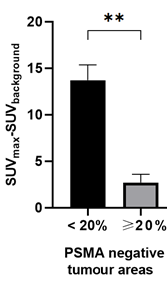


**Figure S4:** **Correlation of SUV_max_-SUV_background_ with growth pattern and PSMA negative tumour areas.**

Significant higher SUV_max_ minus SUV_background_ value were seen in prostate cancers with an expansive growth pattern (p<0.01, Mann-Whitney U test) **(A)** and PSMA IHC negative tumour areas (PSMA_%neg_) < 20% (p<0.01, Mann-Whitney U test) **(B)**.


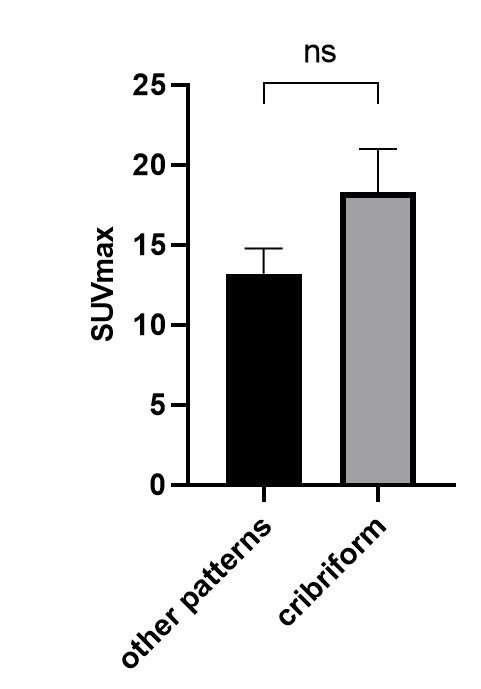


**Figure S5:** **Correlation of prostate carcinomas containing cribriform growth pattern with SUV_max_ values.**

A trend, but no significant difference in SUV_max_ values could be found in cases showing a cribriform growth pattern (p=0.059, Mann-Whitney U test).


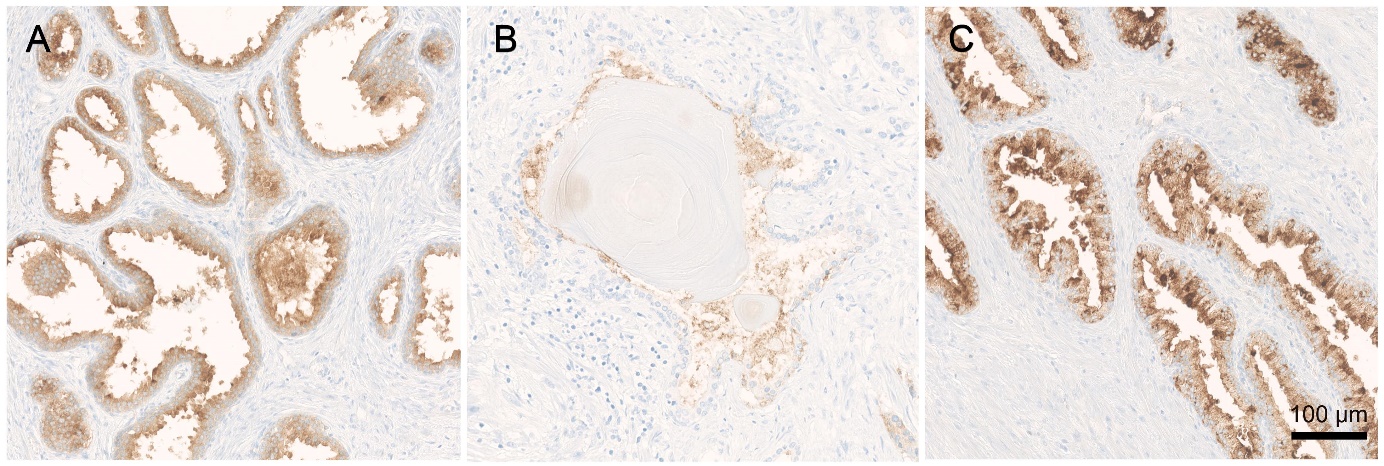


**Figure S6:** **PSMA IHC expression of benign glands in cases with prostate carcinoma showing more than 20% PSMA IHC negative tumour areas and unexpected high SUV_max_ values:**

PSMA expression of benign glands in cases with prostate cancer showing PSMA negative areas of more than 20% but an unexpected high SUV_max_ in the PSMA-PET. All benign glands **(A-C)** show a heterogenous cytoplasmic (1-2+) and membranous (0-1+) expression.
